# Alzheimer’s Disease Genetic Influences Impact the Associations between Diet and Resting-State Functional Connectivity: A Study from the UK Biobank

**DOI:** 10.3390/nu15153390

**Published:** 2023-07-30

**Authors:** Tianqi Li, Auriel A. Willette, Qian Wang, Amy Pollpeter, Brittany A. Larsen, Parvin Mohammadiarvejeh, Mohammad Fili

**Affiliations:** 1 Genetics and Genomics Interdepartmental Graduate Program, Iowa State University, 1109 HNSB, 2302 Osborn Drive Ames, Ames, IA 50011, USA; 2 Department of Neurology, Roy J. and Lucille A. Carver College of Medicine, University of Iowa, Iowa City, IA 52242, USA; aurielwillette@gmail.com; 3 Department of Food Science and Human Nutrition, College of Human Sciences, Iowa State University, Ames, IA 50011, USA; wangqian@iastate.edu; 4 Bioinformatics and Computational Biology Graduate Program, Department of Biomedical Sciences, College of Veterinary Medicine, Iowa State University, Ames, IA 50011, USA; amyrp@iastate.edu; 5 Neuroscience Graduate Program, Department of Biomedical Sciences, College of Veterinary Medicine, Iowa State University, Ames, IA 50011, USA; balarsen@iastate.edu; 6 Department of Industrial and Manufacturing Systems Engineering, College of Engineering, Iowa State University, Ames, IA 50011, USA; pmohamm@iastate.edu (P.M.); mfili@iastate.edu (M.F.)

**Keywords:** fMRI, Alzheimer’s disease, neural network connectivity, red wine consumption, dairy consumption

## Abstract

Background: Red wine and dairy products have been staples in human diets for a long period. However, the impact of red wine and dairy intake on brain network activity remains ambiguous and requires further investigation. Methods: This study investigated the associations between dairy and red wine consumption and seven neural networks’ connectivity with functional magnetic resonance imaging (fMRI) data from a sub-cohort of the UK Biobank database. Linear mixed models were employed to regress dairy and red wine consumption against the intrinsic functional connectivity for each neural network. Interactions with Alzheimer’s disease (AD) risk factors, including apolipoprotein E4 (APOE4) genotype, TOMM40 genotype, and family history of AD, were also assessed. Result: More red wine consumption was associated with enhanced connectivity in the central executive function network and posterior default mode network. Greater milk intake was correlated with more left executive function network connectivity, while higher cheese consumption was linked to reduced posterior default mode network connectivity. For participants without a family history of Alzheimer’s disease (AD), increased red wine consumption was positively correlated with enhanced left executive function network connectivity. In contrast, participants with a family history of AD displayed diminished network connectivity in relation to their red wine consumption. The association between cheese consumption and neural network connectivity was influenced by APOE4 status, TOMM40 status, and family history, exhibiting contrasting patterns across different subgroups. Conclusion: The findings of this study indicate that family history modifies the relationship between red wine consumption and network strength. The interaction effects between cheese intake and network connectivity may vary depending on the presence of different genetic factors.

## 1. Introduction

Dementia arises due to damage or loss of neurons and their connections in the brain, resulting in an irreversible and progressive decline in cognitive function [1]. The worldwide prevalence of dementia is expected to increase significantly, from 57.4 million cases in 2019 to 152.8 million by 2050 [2]. Alzheimer’s disease (AD), which accounts for 60–80% of all dementia cases, is the primary cause of this debilitating condition [3].

Evidence suggests that dietary habits and specific nutrients may influence the progression of cognitive impairment and dementia [4]. Longitudinal studies discovered associations between certain nutrients or dietary patterns and reductions in brain volume [5] or alterations in brain integrity [6]. The association between dairy and red wine consumption and cognitive dysfunction or dementia remains a subject of debate and uncertainty. Further large-scale studies are necessary to clarify this relationship.

One study [7] found that participants aged 60 years and older who consumed high amounts of milk and dairy products experienced a reduced incidence of Alzheimer’s disease dementia. Conversely, another study [8] reported that participants aged 45–64 years who drank more than one glass of milk daily showed a faster cognitive decline compared to those who rarely consumed milk. A recent study suggested that dairy consumption might be linked to an increased risk of Parkinson’s disease [9]. Another study indicated that higher milk and dairy product consumption could be associated with greater cognitive decline [10]. Two recent systemic reviews suggested that the relationship remains inconclusive due to small sample sizes in some studies [11,12].

Research involving participants from Australia [13], the Netherlands [14], and Canada [15] has suggested that fermented dairy products may have preventive effects against dementia. Previous research from our laboratory, using UK data, indicated that adding cheese to the diet daily on a weekly basis is associated with better fluid intelligence performance, in a risk status-dependent manner [16]. However, a large-scale Japanese study found no evidence to suggest that dairy intake was associated with functional disability among older Japanese adults [17].

The relationship between red wine consumption and cognitive function is complex, controversial, and uncertain [18]. Some studies have discovered the role of red wine and dietary grape polyphenols in the prevention and treatment of AD [18,19]. Our prior research demonstrated that, depending on risk status, incorporating red wine into the diet daily on a weekly basis may enhance long-term cognitive outcomes in terms of Fluid Intelligence [16].

It is also important to note that excessive alcohol consumption is a well-established risk factor for multiple chronic diseases [19,20]. Evidence also suggests that chronic alcohol intake is linked not only to cardiac and liver problems but also to cognitive impairments and brain damage [21]. One study indicated that consuming fewer than four drinks per day for women and fewer than eight drinks for men is associated with a reduced likelihood of cognitive impairment compared to abstinence, after accounting for potential confounding factors [22].

Resting-state functional magnetic resonance imaging (fMRI) can forecast cognitive or emotional behavior [23] and identify early alterations in brain activity associated with AD-related cognitive decline [24]. Independent component analysis investigations have revealed discrepancies in this pattern for individuals with mild cognitive impairment (MCI) or AD [25]. Our prior research established a connection between AD risk factors, cognitive impairment, and fMRI findings through the assessment of network connectivity [26,27].

To further investigate the relationship between dairy and red wine consumption and cognitive dysfunction, we analyzed data from a sub-cohort of 12,661 UK Biobank participants and examined the associations between red wine/dairy intake and the connectivity of seven specific cognitive- and emotional-processes-related neural networks, considering various AD risk factors of APOE4 and TOMM40 genotypes and AD family history.

## 2. Materials and Methods

### 2.1. Cohort and Participants

The UK Biobank is a prospective study, including about half a million participants aged 40–70 years, from 22 assessment centers in the United Kingdom [28]. The current study analyzed a sub-cohort of 12,661 participants with genomics, MRI, diet, and demographics data. In order to reduce the influence of diseases on neural network outcomes, we excluded 636 participants who had neurological disorders, as determined by ICD-10 codes. Specifically, individuals who had central nervous system diseases (G00–G99), cerebrovascular diseases (I60–I69), or mental and behavioral disorders (F00–F99) were excluded (Supplementary Figure S1). There were 2715 valid cases for the red wine analysis and 636 ones for milk consumption, with cheese being 5171.

### 2.2. Resting-State fMRI

Participants underwent scanning at one of three locations (Reading, Newcastle, or Manchester) using a Siemens Skyra 3T unit equipped with a 32-channel RF receiver head coil (Siemens Medical Solutions, Erlangen, Germany) [29]. Baseline MRI assessments started in 2014, with continuous longitudinal data collection [30]. During scanning, participants were instructed to keep their eyes open, concentrate on a crosshair, and avoid specific thoughts. Each scan lasted 6 min and 10 s, acquiring 490 images with particular acquisition parameters. The UK Biobank white papers (https://biobank.ctsu.ox.ac.uk/crystal/crystal/docs/brain_mri.pdf (accessed on 29 June 2023)) detail preprocessing and quality control procedures. In summary, FSL tools were utilized to motion-correct, grand-mean intensity normalize, high-pass temporal filter (with sigma = 50.0 s), and unwarp and denoise EPI and GDC (using ICA + FIX processing) the 4D dataset. FMRIB’s MELODIC was employed for group principal component analysis and independent component analysis, resulting in spatially orthogonal, non-noise, distinct independent components (ICs) representing resting neural networks [31]. The Papaya viewer allows for online visualization of these ICs: see https://www.fmrib.ox.ac.uk/ukbiobank/group_means/rfMRI_ICA_d25_good_nodes.html (accessed on 29 June 2023) for a link to the viewer and maps.

An IC was spatially back-projected onto their EPI scan to calculate intrinsic functional connectivity for each participant. The average activation level was determined from the initial T-value map and converted to a Z-score for simpler interpretation. This average activation level was subsequently used in statistical analyses. As described in our previous study, an expert (AAW) examined the activation maps and characterized the neural networks [26]. The present study focused on seven cognitive- and emotional-functions-related networks (see Supplemental Table S1).

### 2.3. Genetic Factors—APOE, TOMM40, and Family History

Genotyping was performed with the UK BiLEVE Axiom or UK Biobank Axiom array [32]. The APOE haplotype isoforms (ε2, ε3, and ε4) were identified using SNPs rs429358 and rs7412. Participants were categorized as either ε4 non-carriers or carriers based on whether participants had at least one ε4 allele (ε2/ε4, ε3/ε4, and ε4/ε4) or none (ε2/ε2, ε2/ε3, and ε3/ε3).

As described in our previous article, TOMM40 genotype data for the SNP ‘650 were extracted for analyses using PLINK version 1.90 (https://www.cog-genomics.org/plink/1.9/ (accessed on 29 June 2023)). TOMM40 ‘650 status was coded as those who were non-G carriers (AA homozygotes) versus G-carriers (GA and GG) [27].

AD family history classification relied on participants’ self-reported presence or absence of AD in their family history via the touchscreen questionnaire. Participants were asked about their family history through the question, “Has/did your father/mother ever suffer from:”, which was followed by a list of chronic diseases, including ‘Alzheimer’s disease/dementia’.

### 2.4. Covariates

Covariates in the analysis consisted of baseline age (in years) and sex (male or female). Additional covariates included alcohol consumption status, smoking status, body mass index (BMI), and the Townsend Index (for social-economic stratification). Alcohol consumption status was classified as never, previous, or current drinker, while smoking status was categorized as never, former, or current smoker [27]. The Townsend deprivation index was calculated before each participant joined the UK Biobank based on the national census output areas. The participants were assigned a score that corresponded to their postcode’s output area.

### 2.5. Diet Consumption

Participants were prompted to provide information regarding their dietary habits, including the consumption of food and alcoholic beverages, through an interactive touchscreen questionnaire. A particular focus was placed on their 24 h intake of red wine, categorized by distinct varieties. Moreover, the examination employed a 24 h dietary recall method to assess the consumption of milk and cheese. Milk intake was documented in daily 250 m increments (glass equivalents). As for cheese consumption, individuals were inquired about their intake the previous day, including instances in sandwiches, atop burgers or jacket potatoes, or incorporated into pasta dishes. Cheese servings were quantified through descriptors such as “a small matchbox-sized portion”, “a large spoonful or a handful”, or “an amount sufficient to cover a standard square sandwich bread slice”.

### 2.6. Statistical Analyses

Data preparation and analyses were completed by using R, version 4.2.0 (RStudio, Posit Software, Boston, MA, USA) and SPSS 26 (IBM Corp., Armonk, NY, USA). Linear mixed models were used to regress predictors of diet consumption against each resting state IC.

The initial analysis examined the primary effects of red wine, milk, and cheese consumption on each neural network, accounting for previously mentioned covariates. Additionally, potential interactions with family history, TOMM40, and APOE4 status were investigated to determine whether these factors moderated the associations between dietary intake and neural network connectivity strength. The assessment centers where participants consented were served as the random effects. A significance level of *p* < 0.05 was employed for main effects, while an alpha level of 0.10 was used for interaction effects to offset potential reductions in statistical power [33,34]. The omnibus MANCOVA testing was first done to limit type 1 error [35]. Interventionary studies involving animals or humans, and other studies that require ethical approval, must list the authority that provided approval and the corresponding ethical approval code.

## 3. Results

### 3.1. Demographics and Data Summaries

Demographics and baseline characteristics are listed in Table 1. The Supplementary Table S1 lists and describes all seven non-noise derived cognitive- and emotional-processes-related neural networks.

### 3.2. Main Effects

**Figure 1:**
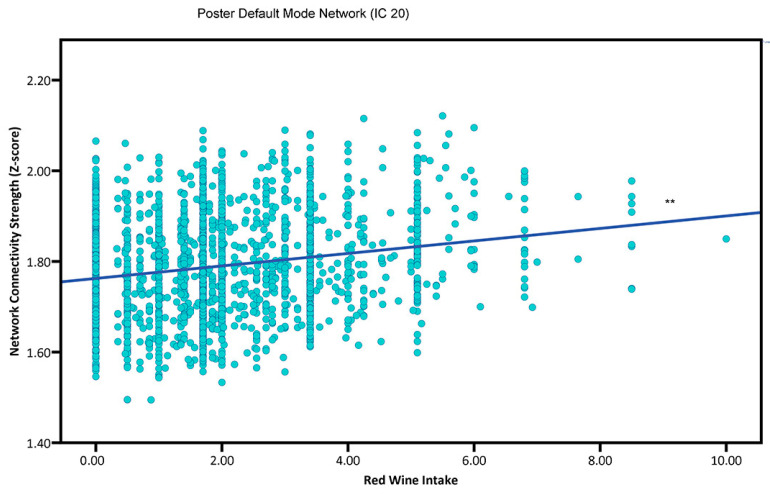
The association between red wine consumption and intrinsic functional connectivity (i.e., neural network activity) in adults. ** *p* < 0.01.

More red wine consumptions were associated with greater central executive function network (IC 9; β = 0.0212, SE = 0.0085, *p* = 0.0128) and posterior default mode network (IC 20; β = 0.0182, SE = 0.0059, *p* = 0.0021) (Figure 1) connectivity.

More milk intake was associated with greater left executive function network connectivity (IC 13; β = 0.0314, SE = 0.0143, *p* = 0.0287). However, more cheese intake was associated with less posterior default mode network (IC 20; β = −0.0241, SE = 0.0094, *p* = 0.0109) connectivity.

### 3.3. Red Wine Consumption by Family History Interactions

**Figure 2:**
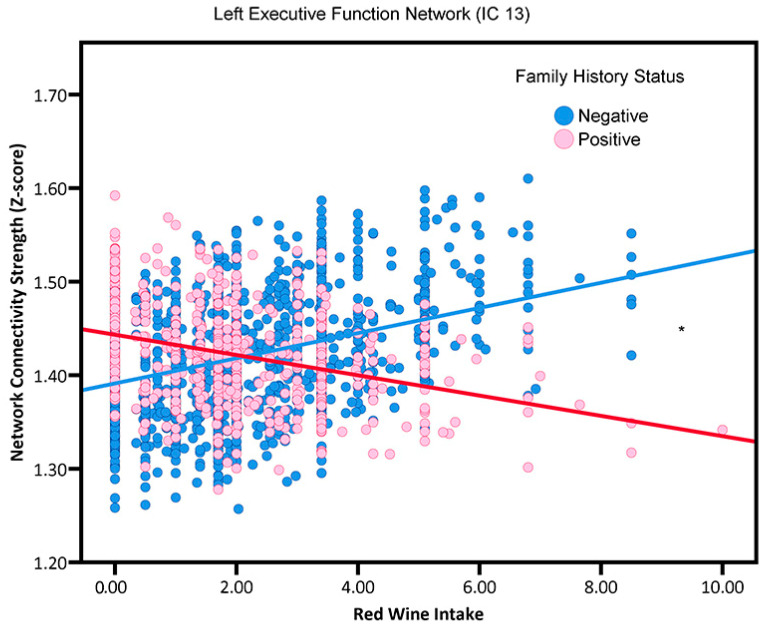
The association between red wine consumption and left executive function network (i.e., neural network activity) in adults without or with a family history of AD (“positive”, “negative”). Blue circles and red stars, respectively represent family history negative and APOE4 positive participants. * *p* < 0.05.

Increased red wine consumption was found to be positively correlated with enhanced connectivity in the left executive function network (IC 13; β = 0.0135, SE = 0.0009, *p* = 0.0000) (Figure 2) for participants without a family history of AD. Conversely, participants with a family history of AD exhibited reduced network connectivity in relation to their red wine consumption.

### 3.4. Cheese Intake by APOE4 Status, TOMM40 Status, and Family History Interactions

The relationship between cheese consumption and neural network connectivity was strongly influenced by APOE4 status, TOMM40 status, and family history, with the associations displaying contrasting tendencies across different subgroups. In general, increased cheese intake was associated with reduced neural network connectivity in participants without genetic risk factors (negative APOE4 status, TOMM40 status, and family history). Conversely, participants who were APOE4 and TOMM40 carriers or had a positive family history of Alzheimer’s disease exhibited a positive association between cheese consumption and neural network connectivity.

**Figure 3:**
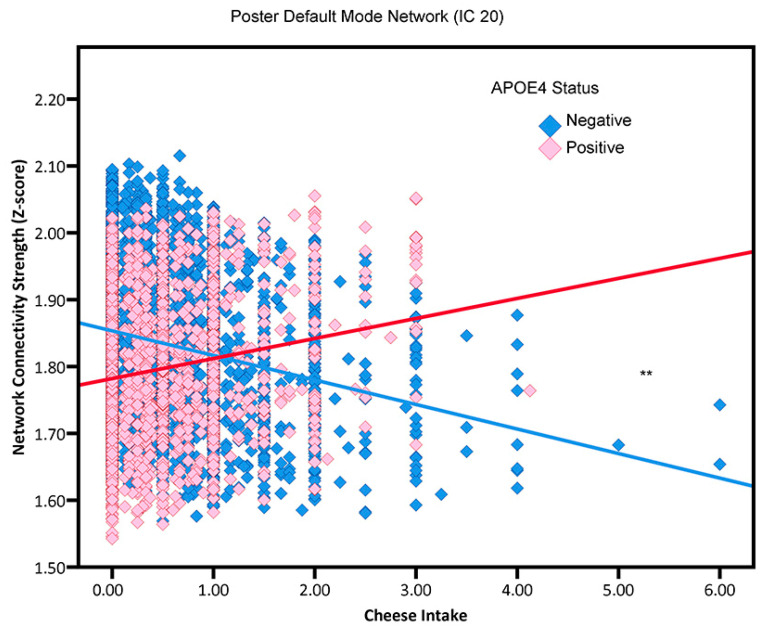
The association between cheese consumption and posterior default mode network (i.e., neural network activity) in adults without or with an APOE4 genotype (“positive”, “negative”). Blue squares and red squares, respectively represent APOE4 negative and APOE4 positive participants. ** *p* < 0.01.

More cheese consumption was correlated with reduced network connectivity in executive function networks (IC 5, IC 9, and IC 13), affect processing network (IC 10), fronto-cingular network (IC 14), and posterior default mode network (IC 20) (Figure 3) for non-APOE4 carriers. In contrast, among APOE4 carriers, greater cheese intake was positively associated with enhanced network connectivity in executive function networks (IC 5, IC 9, and IC 13), affect processing network (IC 10), fronto-cingular network (IC 14), and posterior default mode network (IC 20) (see Table 2).

**Figure 4:**
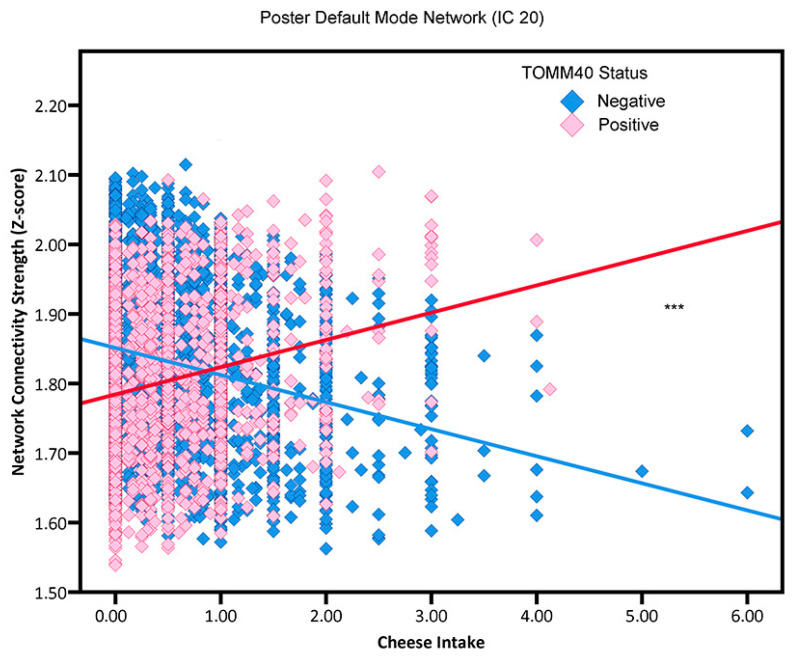
The association between cheese consumption and posterior default mode network (i.e., neural network activity) in adults TOMM40 650′ genotype (“positive”, “negative”). Blue squares and red squares, respectively represent TOMM40 650′ G non-carriers and TOMM40 650′ G carriers. *** *p* < 0.001.

Regarding TOMM40 status, the associations mirrored those observed for APOE4 status. For non-TOMM40 carriers, increased cheese consumption was correlated with reduced network connectivity in executive function networks (IC 5 and IC 9), affect processing network (IC 10), fronto-cingular network (IC 14), and posterior default mode network (IC 20). In contrast, among TOMM40 carriers, greater cheese intake was positively associated with enhanced network connectivity in executive function networks (IC 5 and IC 9), affect processing network (IC 10), fronto-cingular network (IC 14), and posterior default mode network (IC 20) (refer to Table 2; Figure 4).

In terms of family history, the pattern was similar to the findings for APOE4 and TOMM40 status, albeit with fewer significant networks. Among participants without a family history of Alzheimer’s disease, increased cheese consumption was associated with reduced network connectivity in the anterior and posterior default mode network (IC 1), executive function networks (IC 5 and IC 13), and fronto-cingular network (IC 14). Conversely, for participants with a positive family history, greater cheese intake was linked to more network connectivity in the anterior and posterior default mode network (IC 1), executive function networks (IC 5 and IC 13), and fronto-cingular network (IC 14).

## 4. Discussion

The aim of this study was to investigate the associations between red wine and dairy consumption and seven distinct cognitive- and emotional-processes-related neural network connectivity in participants, considering the influence of TOMM40 and APOE4 carrier status and family history.

### 4.1. Red Wine

Our primary findings revealed that, for the main effect test, increased red wine consumption correlated with enhanced central executive function network and posterior default mode network connectivity.

The central executive function network is responsible for managing and manipulating information in working memory, as well as facilitating decision-making and problem-solving for goal-oriented behavior [36]. Studies have indicated that, during early adolescence, the neural architecture of both the central executive function network and default mode network undergoes changes, resulting in the fine-tuning of within-network and between-network connectivity [37]. Enhanced connectivity between these two networks has been linked to improved social skills at the metacognitive abilities level [38]. Therefore, the consumption of grape polyphenols found in dietary sources, such as red wine, may have a protective effect against the deterioration of metacognitive abilities.

We also found that, for participants with a family history of AD, the relationship between red wine consumption and left executive function network activity exhibits differently compared to those without a family history of AD. Red wine consumption exhibited a beneficial impact on participants without a family history of AD, while no such effect was observed among those with a positive family history.

Alcohol is known to have adverse effects on various chronic diseases, such as cardiovascular diseases, diabetes, liver cirrhosis, and even cancer, and it is also considered to have neurotoxic effects on the brain [39]. Furthermore, alcohol has been recognized as a risk factor for dementia and cognitive decline [40], with a significant role in the development of early onset dementia [41]. On the other hand, research also has suggested a U-shaped relationship between alcohol consumption and cognitive function scores, where low to moderate alcohol intake is associated with better global cognition scores [40,42]. The positive effects of alcoholic beverages, such as wine, on reducing the risk of cardiovascular and cerebrovascular diseases have been partially attributed to alterations in lipid profiles and factors related to blood flow and hemostasis [43]. Numerous studies have reported that wine consumption, in particular, is linked to a reduced risk of developing dementia, specifically Alzheimer’s disease [43]. The protective associations identified for wine might be attributable to components other than ethanol. Wine is considered a dietary source of phytochemicals, with red wine being particularly rich in a wide array of polyphenolic compounds that could exhibit neuroprotective activities [18]. Several aglycone forms of polyphenols have been investigated as potential novel dietary or supplemental strategies for the prevention and/or treatment of Alzheimer’s disease dementia and associated brain pathology recently [43]. In line with other research [42], our findings also indicate that this protective effect may be influenced by genetic factors, family history. However, we did not observe significant differences between various APOE4 or TOMM40 genotypes.

### 4.2. Dairy Consumption

Our results suggested greater milk intake was linked to augmented left executive function network connectivity for the main effects. In contrast, greater cheese consumption was found to be negatively correlated with posterior default mode network connectivity. No significant differences were found between AD risk groups (APOE4 and TOMM40 genotypes and family history) regarding the interaction test for milk intake. However, for cheese consumption interactions, participants with genetic risk factors (APOE4 and TOMM40 genotypes and family history) displayed a strong association between cheese consumption and the connectivity of most neural networks.

The association between milk consumption and executive function network remains ambiguous. While some research has indicated a connection between the overall intake of dairy products and executive and cognitive functions, a direct relationship between milk consumption and these functions has not been consistently established [15,44]. Nevertheless, a 17-year longitudinal study conducted with an older population found that milk consumption exhibited a linear protective effect against dementia [7]. One study has reported that dairy products, excluding milk intake, were associated with the executive function domain [15]. In contrast, other research has observed a significant linear trend for cognitive outcome scores across increasing dairy food intake groups, as documented in the National Health and Nutrition Examination Survey’s investigation of the association between dairy product consumption and cognitive function [44]. Additionally, another study found that milk consumption may be associated with reduced cognitive impairment, as assessed by the Mini-Mental State Examination [10]. L. C. de Goeij et al. suggested that skimmed milk and buttermilk intake are significantly associated with better executive functioning [14]. Our data suggested that, generally, milk consumption has a benefit effect on neural network connectivity.

The effects of cheese intake on cognitive outcomes remain inconclusive. A South Australia-based study suggested that cheese consumption was linked to negative cognitive outcomes [13]. Researchers from eastern Finland found that high saturated fat intake from dairy products correlated with poor cognitive function and an increased risk of mild cognitive impairment [45]. A 13-year follow-up study of elderly French women suggested cognitive decline was associated with higher intakes of dairy desserts [46]. Meanwhile, studies from Korea [15], Spain [47], Maine State [44], and France [48] reported no associations between cheese or fermented dairy consumption and changes in cognitive performance [47]. However, other studies from Finland [49] and Spain [10] have indicated that cheese may have beneficial effects. Additionally, research from Japan [7], Canada [15], Singapore [50], and the Netherlands [14] support positive associations between the consumption of fermented dairy products and cognitive function assessments.

In our previous study, we observed that family history moderated the positive association between cheese intake and cognitive trajectories [16]. In the current study, we employed a different measure of cheese consumption. While our previous study relied on a week recall, this time we utilized a more accurate twenty-four-hour recall and incorporated data from five measurement cycles. We observed a substantial influence of AD risk factors on the associations between cheese consumption and neural network connectivity in the present study. Notably, our findings revealed that individuals with AD risk factors exhibited a highly significant association between cheese consumption and the connectivity of most cognitive-related neural networks.

Research investigating the associations between dairy consumption and cognitive performance is still in its infancy, and the underlying mechanisms and effects have yet to be fully elucidated [14]. Certain components and nutrients in dairy products may promote healthy brain function during aging. For instance, some bioactive peptides [10,51] could be beneficial for maintaining healthy brain function as we age. Another study suggested that novel lactopeptides derived from digested fermented dairy products may help prevent age-related cognitive decline [52]. Evidence also indicates that the beneficial effects may stem from milk fat, calcium, magnesium, potassium, or a combination of these nutrients as part of the unique package that dairy provides [53]. However, these positive effects could be moderated by other factors [54].

Our findings suggest that AD genetic risk factors play a significant role. Family history is a particularly interesting factor. Apart from genetics, it may be related to dietary habits and gut microbiota. Preclinical studies have shown that the probiotic effects of lactic acid bacteria can reduce pro-inflammatory cytokines, decrease oxidative stress, and increase brain-derived neurotrophic factors, potentially promoting neuronal growth and survival [55,56].

Several limitations in our study should be acknowledged. The red wine and dairy consumption data were obtained from the UK Biobank and are based on self-reported dietary information, which can introduce recall bias and inaccuracies. Although we employed more accurate assessments, using objective biomarkers of dietary intake or conducting experiments could further enhance the validity of our findings. While we controlled for several potential confounders, there may be other unmeasured factors that could influence the observed associations, such as physical activity and overall diet quality. The UK Biobank may not be representative of the broader diverse population, limiting the generalizability of our findings. Future studies should aim to include more diverse populations in terms of ethnicity to better understand the associations under investigation.

By addressing these limitations, future research can be conducted using more diverse, large-scale databases to investigate genetic variations that may contribute to the associations between dairy and red wine consumption, AD risk factors, and cognitive performance. This would provide a more comprehensive understanding of the relationships between diet, genetics, and cognitive health across different populations, and ultimately contribute to the development of evidence-based dietary recommendations and interventions aimed at preserving cognitive function and reducing the risk of Alzheimer’s disease and other age-related cognitive impairments.

## 5. Conclusions

In summary, our study provides valuable insights into the associations between dairy and red wine consumption, AD risk factors, and cognitive performance in relation to neural network connectivity. Our findings suggest that low to moderate red wine consumption may have a protective effect on cognitive performance, particularly among individuals without a family history of AD. The associations between cheese consumption and neural network connectivity were found to be influenced by genetic risk factors, highlighting the importance of considering genetic variability in future studies. However, the effects of milk and cheese intake on cognitive performance remain inconclusive, warranting further investigation.

## Figures and Tables

**Table 1:** Characteristics of participants.

Characteristic		
Baseline Age, mean (SD), y	55.07 (7.48)	Range: 40–70
Body mass index (BMI), mean (SD), kg/m^2^	26.59 (4.17)	Range: 14.74–56.12
Female, %	52.54	
APOE ε4 Status, %	27.68	
TOMM40 ‘650 status, %	26.57	
Family History of AD, %	24.26	
Smoking Status, %		
Never	60.74	
Previous	32.89	
Current	6.37	
Alcohol Status, %		
Never	2.45	
Previous	1.95	
Current	95.60	
Milk, mean (SD), glasses/carton//250 mL	1.48 (1.06)	Range: 0.5–5
Cheese, mean (SD), servings	0.52 (0.62)	Range: 0–6
Red wine, mean (SD), glasses/carton//250 mL	1.40 (1.02)	Range: 0.5–5

AD = Alzheimer’s disease; APOE = apolipoprotein E. TOMM40 = translocase of outer mitochondrial membrane 40. All measures were obtained at baseline with the exception of milk consumption, cheese consumption, and red wine consumption (collected over five visits and averaged).

**Table 2:** Estimates by group for cheese intake with AD risk factors.

Component	APOE Status				TOMM40 Status				Family History Status			
	APOE4 Negative		APOE4 Positive		TOMM40 650′ Negative		TOMM40 650′ Positive		Negative		Positive	
	Beta	SE	Beta	SE	Beta	SE	Beta	SE	Beta	SE	Beta	SE
IC 1	−0.0190	0.0038	0.0320	0.0063	−0.0190	0.0038	0.0320	0.0063	**−0.0244**	0.0038 *	**0.0553**	0.0059 *
IC 5	**−0.0138**	0.0025 **	**0.0632**	0.0042 **	**−0.0179**	0.0025 ***	**0.0774**	0.0044 ***	**−0.0104**	0.0026 *	**0.0616**	0.0040 *
IC 9	**−0.0418**	0.0034 **	**0.0695**	0.0056 **	**−0.0398**	0.0034 **	**0.0683**	0.0057 **	−0.0213	0.0034	0.0176	0.0055
IC 10	**−0.0340**	0.0038 **	**0.0621**	0.0063 **	**−0.0349**	0.0037 **	**0.0685**	0.0065 **	−0.0170	0.0038	0.0198	0.0064
IC 13	**−0.0089**	0.0018 **	**0.0540**	0.0030 **	−0.0029	0.0018	0.0391	0.0031	**−0.0045**	0.0018 *	**0.0475**	0.0029 *
IC 14	**−0.0141**	0.0021 *	**0.0234**	0.0035 *	**−0.0148**	0.0021 *	**0.0265**	0.0035 *	**−0.0165**	0.0021 **	**0.0357**	0.0032 **
IC 20	**−0.0367**	0.0028 **	**0.0300**	0.0047 **	**−0.0389**	0.0028 ***	**0.0392**	0.0049 ***	−0.0301	0.0029	0.0169	0.0045

Bolded text denotes *p* < 0.05. Neural networks with significant interactions * *p* < 0.05, ** *p* < 0.01, *** *p* < 0.001.

## Data Availability

The data in this study are owned by the UK Biobank (https://www.ukbiobank.ac.uk/ (accessed on 29 June 2023)), a data repository that can be accessed by applying through the UK Biobank Access Management System (https://www.ukbiobank.ac.uk/enable-your-research/register (accessed on 29 June 2023)). Due to the legal agreement, as researchers, we do not have permission to share the data, and we are not entitled to republish or otherwise make available any UK Biobank data at the individual participant level. All analyses and intellectual content separate from UK Biobank are available on a request basis.

## Supplementary Materials

The following supporting information can be downloaded at: https://www.mdpi.com/article/10.3390/nu15153390/s1, Figure S1: Flowchart Diagram of Participant Selection and Exclusion; Table S1: Interpretation of Independent Components that Constitute Neural Networks in UK Biobank.
