# Supplementary material for: Alzheimer’s Disease Genetic Influences Impact the Associations between Diet and Resting-State Functional Connectivity: A Study from the UK Biobank
Source: Nutrients. 2023 Jul 30;15(15):3390. doi: 10.3390/nu15153390 (PMC10420831; doi:10.3390/nu15153390)
Supplement: Supplementary file 1 [file nutrients-15-03390-s001.zip › nutrients-2509532-supplementary.pdf]

Supplementary Figure S1. Flowchart Diagram of Participant Selection and Exclusion

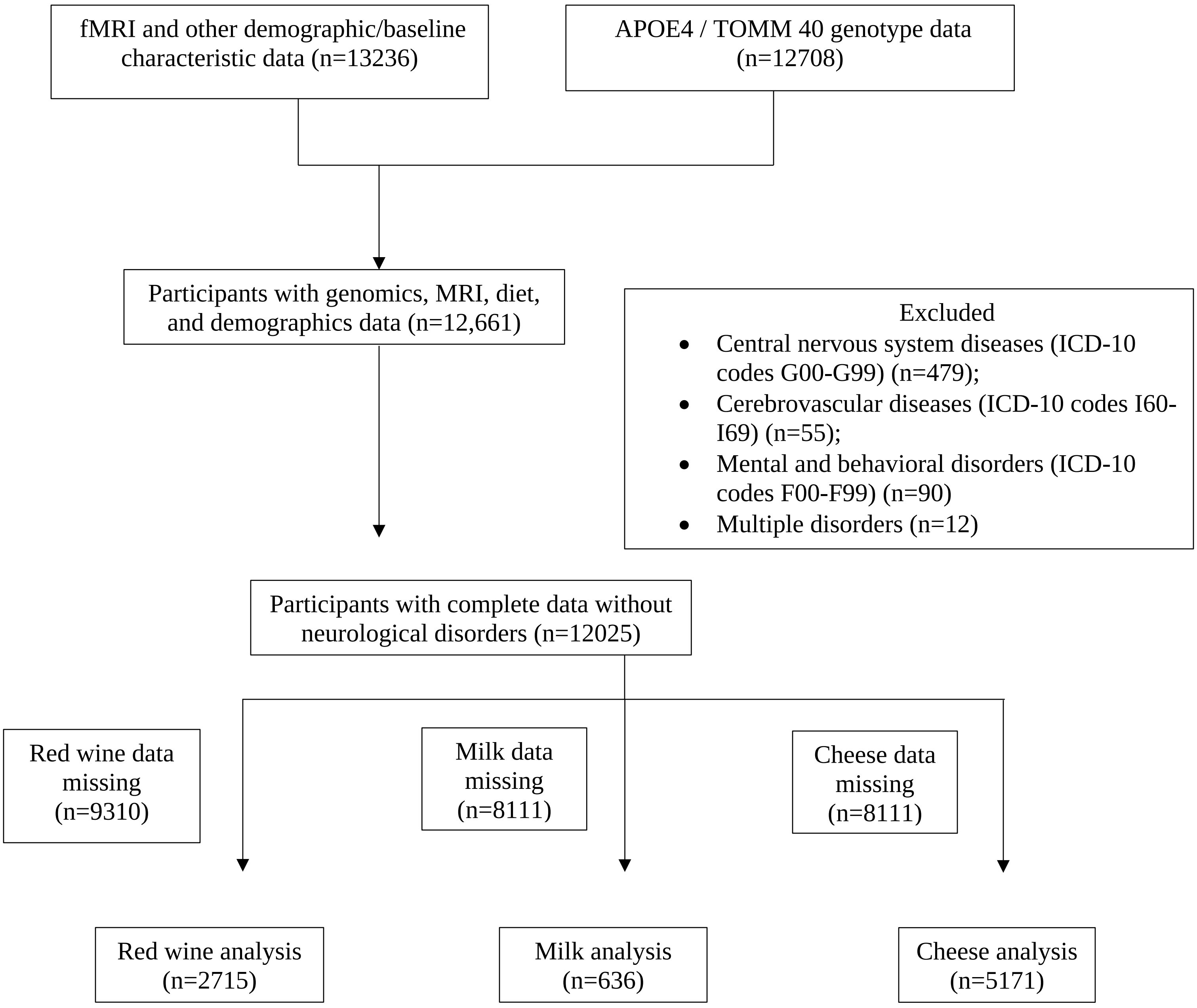

Supplementary Table S1. Interpretation of Independent Components that Constitute Neural Networks in UK Biobank

Independent Component (IC)	Neural Network Description
1	Anterior and Posterior Default Mode Network
5	Right Executive Function Network
9	Central Executive Function Network
10	Affect Processing Network
13	Left Executive Function Network
14	Fronto-Cingular Network
20	Posterior Default Mode Network

IC 1 was “classic” Default Mode Network, with activation in medial prefrontal cortex and the posterior cingulate/precuneus region. IC 5 was “classic” executive network restricted to the right hemisphere, composed of inferior frontal and parietal gyri. IC 9 was “classic” central executive network and composed of bilateral inferior frontal and parietal gyri. IC 10 had a contiguous region in either hemisphere comprising operculum, primary sensory and motor cortices, mid-frontal gyrus, and anterior insula, which may represent synthesis of internal states to give rise to emotional processing and interpretation. IC 13 was a left executive function network comprised of inferior and parietal gyri. IC 14 was a fronto-cingular network composed of all segments of cingulate gyrus except for the rostrum, as well as medial frontal gyrus. IC 20 comprised the posterior portion of Default Mode Network.
